# Treatment outcomes in relapsed acute promyelocytic leukemia patients initially treated with all-*trans* retinoic acid and arsenic compound-based combined therapies

**DOI:** 10.3892/ol.2013.1643

**Published:** 2013-10-25

**Authors:** JIN LU, XIAOJUN HUANG, LI BAO, HAO JIANG, HONGHU ZHU, BIN JIANG

**Affiliations:** Institute of Hematology, Peking University People’s Hospital, Beijing 100044, P.R. China

**Keywords:** acute promyelocytic leukemia, relapse, re-induction therapy, remission, all-*trans* retinoic acid, arsenic compound

## Abstract

Contemporary combined therapies that include the use of all-*trans* retinoic acid (ATRA) and arsenic compounds have reduced relapse rates from ~50 to <10% in acute promyelocytic leukemia (APL) patients, however relapse treatment remains controversial. Treatment outcomes in relapsed patients with APL previously treated with combined ATRA + arsenic compound therapy were investigated. A retrospective, observational study was conducted of 25 patients with APL (male to female ratio, 17:8; mean age, 36.4±10.3 years) exhibiting first-time relapse following combined ATRA + arsenic compound therapy. These patients were subsequently treated with secondary ATRA + arsenic compound therapy, salvage chemotherapy, monoclonal antibody therapy or intrathecal chemotherapy, between January 1994 and December 2010. The overall remission rate, duration of remission and toxic effects were assessed. Patient outcomes included mortality during secondary induction therapy (6/25, 24.0%); complete recovery from central nervous system (CNS) relapse following intrathecal chemotherapy (1/25, 4.0%); complete remission following ATRA + arsenic compound therapy (10/25, 40.0%), chemotherapy (3/25, 12.0%) and targeted therapy (1/25, 4.0%); and non-remission (NR) following ATRA + arsenic compound therapy (4/25, 16%). Four (16.0%) patients were subsequently treated with allogeneic hematopoietic stem cell transplantation (allo-HSCT), two of which remained disease-free at the end of the study period and two of which succumbed to the disease. Secondary bone marrow and CNS relapse occurred in 14 (56.0%) patients and one (4.0%) patient, respectively. ATRA + arsenic compound-based combination therapy was effective in re-inducing morphological remission in relapsed patients with APL with previous exposure to ATRA + arsenic compounds, producing low molecular remission rates and high risk of secondary relapse. Furthermore, investigation of early allo-HSCT is required to determine its potential as a therapeutic option for re-inducing morphological remission in relapsed patients with APL with previous exposure to ATRA + arsenic compounds.

## Introduction

Acute promyelocytic leukemia (APL) is a relatively rare subtype of acute myelogenous leukemia that occurs in 8–15% of all acute non-lymphoblastic leukemia patients, with a mean incidence of two to three cases per million members of the global population each year (1). APL is characterized by pathological coagulation (coagulopathy) involving abnormal accumulation of immature granulocytes, particularly promyelocytes, leading to fibrinolysis and hemostatic failure (1,2). Unlike other leukemia subtypes, optimal treatment of APL requires rapid initiation of all-*trans* retinoic acid (ATRA) therapy and targeted supportive care for APL-specific complications, including bleeding disorders, APL differentiation syndrome, QT prolongation and other ATRA-related toxicities (3). The wide-spread clinical employment of combined ATRA regimens, including ATRA and arsenic compounds, has reduced relapse from ~50% to <10% in adult patients with APL over the past two decades (4,5). However, increased knowledge of the outcomes in this remaining group of treated patients with APL that exhibit relapse is crucial to understanding APL pathophysiology and to improving survival in this patient subpopulation.

APL is caused by the cumulative effects of somatic mutations, ultimately resulting in the development of mutagen-induced carcinogenesis, and often occurs with advanced age (1). Cytogenetically, between 95 and 100% of APL cases have been reported to be associated with karyotypic abnormalities involving pathognomonic translocations at *t*(15;17)(q22–24;q11–21) that juxtapose the retinoic acid receptor α (RARα) gene with the promyelocytic leukemia (PML) gene (6,7). This translocation has been implicated in the blockage of normal differentiation of immature myeloid cells into mature granulocytes (8,9) and the inhibition of programmed cell death in myeloid cells (7). Furthermore, 10–50% of all patients with APL exhibit FLT3 mutations, either as internal tandem duplications or kinase domain mutations (10), and FLT3 mutations generally correlate with high white blood cell (WBC) counts (>10×10^9^/l), which are indicative of higher patient risk of relapse (6).

ATRA treatments for APL are unique in that they act by dissociating the nuclear hormone receptor complex NCOR-HDAC from RARα. This then initiates the maturation of leukemic promyelocytes, rather than inducing cell death (11). While ATRA monotherapies have demonstrated relatively high relapse rates, combined therapies involving anthracyclines and other active agents are able to markedly reduce relapse rates (12). Arsenic compounds, such as arsenic trioxide (ATO) and arsenic tetrasulfide (ATS), are the most active single agents in refractory APL treatment due to their ability to induce partial myeloid differentiation and caspase-specific apoptosis (13), with a relapse rate of <20% following monotherapy (14). Combined treatment regimens involving both ATRA and ATO have been reported to eradicate leukemic progenitor cells and reduce the relapse rate to <10% in even high-risk patients with APL (15). Furthermore, combined ATRA- and arsenic compound-based (ATRA + arsenic compound) salvage therapies have been demonstrated to induce complete remission in 50–80% of refractory or relapsed patients with APL (16).

Though contemporary combined ATRA + arsenic compound therapies are effective in inducing complete remission (CR) in the majority of patients, a notable patient group still exhibits relapse, although the characteristics of this group are relatively undocumented. For these patients, the risks and benefits of ATRA + arsenic compound retreatment versus retreatment with other modalities remain controversial, as few evidence-based studies have specifically examined cohorts of relapsed patients with APL. The current study examines the characteristics and effectiveness of the treatment of first-time relapsed patients with APL that were originally treated with combined ATRA + arsenic compound therapies. The results provide a unique insight into the outcomes of these patients that is useful for the evaluation and selection of treatment strategies.

## Materials and methods

### Study design

A total of 25 first-time relapse patients with APL, who were previously treated with first-line ATRA + arsenic compound therapy and were subsequently treated in the Hematological Unit of Peking University People’s Hospital (Beijing, China) between January 1994 and December 2010, were included in this retrospective, observational analysis. The study protocol was approved by the Institutional Review Board at Peking University People’s Hospital. All patients provided written informed consent, prior to their treatment, for the use of their data in the subsequent research.

### Patients

Patients were included that: i) were diagnosed with APL in accordance with the morphological criteria (M0–M7) of the French-American-British classification system for myelocytic leukemias (17); ii) exhibited APL confirmed by both cytogenetic assay for *t*(15;17)(q24;q21) and reverse transcription polymerase chain reaction (PCR) analysis for *PML-RARα*, as previously described by de Botton *et al*(18); iii) underwent initial induction therapy with first-line ATRA (25 mg/m^2^/day) and low-dose cytotoxic agents, with or without adjuvant treatment with ATO (10 mg/kg/day) or up-titrated ATS (2250–60 mg/kg/day); iv) exhibited relapse, defined as any disease recurrence following CR, including morphological, molecular and extra-medullary relapses; and v) were treated with consolidation chemotherapy involving cytarabine (Ara-C)- or anthracycline-based therapies with alternating maintenance ATRA (25 mg/m^2^/day) for two weeks, once every three months, and ATO (10 mg/kg/day) or ATS (60 mg/kg/day) for two weeks, twice every three months. WBC and platelet counts were further used to classify patients as low risk (<10×10^9^/l; >40×10^9^/l), intermediate risk (<10×10^9^/l; <40×10^9^/l) or high risk (≥10×10^9^/l; <40×10^9^/l), respectively, as previously described (18).

### Relapse and re-induction therapy

Following confirmation of APL relapse, appropriate re-induction regimens were immediately administered using one of six therapeutic regimens: i) ATRA + arsenic compound combination therapy (Ruijin Pharmaceuticals Co., Ltd., Shanghai, China); salvage chemotherapy with ii) mitoxantrone (Shenghe Pharmaceuticals Co., Ltd., Sichuan, China) + Ara-C (Pfizer, Inc., New York, NY, USA) (MA), iii) homoharringtonin (Minsheng Pharmaceutical Group Co., Ltd., Zhejiang, China) + Ara-C (HA), or iv) homoharringtonin + Ara-C + daunomycin (Pfizer, Inc.) (HAD); v) gemtuzumab ozogamicin treatment (Wyeth Pharmaceuticals, Philadelphia, PA, USA); or vi) intrathecal chemotherapy with cytarabine and dexamethasone (CSPC Zhongnuo Pharmaceutical Co., Ltd., Shijiazhuang, China). Relapse types were classified as morphological relapse (≥5% blasts per abnormal promyelocytes in the bone marrow or per leukemic cells in the peripheral blood), molecular relapse (*PML/RARα* gene conversion from PCR-negative to -positive in patients without morphological abnormalities in two successive four-week bone marrow samples) or extramedullary relapse (abnormal promyelocytes in the cerebrospinal fluid or extramedullary granulocytic sarcoma).

### Laboratory monitoring and assessments

Follow-up bone marrow aspiration was repeated at three-month intervals during maintenance therapy (ATRA + arsenic compounds with alternating maintenance chemotherapy) administration. Patient tolerance, based on gastrointestinal reactions and hepatotoxicity (reduced drug dose when hepatotoxicity grade ≥3 and drug withdrawal when hepatotoxicity grade 4), and urine arsenic compounds were closely monitored, and the doses of arsenic compounds were adjusted in accordance with standards published by the National Cancer Institute (19).

### Outcome assessments

The patients were followed up for a minimum of six months after relapse treatment. The outcome of post-retreatment remission rates, duration of remission and toxic effects were recorded. CR was defined as <5% blasts or abnormal promyelocytes in the bone marrow, coupled with peripheral blood absolute neutrophil count ≥1.5×10^9^/l, untransfused hemoglobin levels ≥100 g/l and platelet count ≥100×10^9^/l. Molecular remission was defined as a negative bone marrow PCR for the *PML*/*RARα* gene at a sensitivity of 10^−4^. Treatment with reconsolidation therapies and other therapies, such as allogeneic and autologous hematopoietic stem cell transplantation (allo-HSCT and auto-HSCT, respectively), were recorded.

### Statistical analysis

This was a retrospective, observational analysis and only descriptive statistics are provided. Data are presented as the mean ± standard deviation, the mean ± interquartile range or the percentile [n (%)], as appropriate.

## Results

### Clinical characteristics of patients initially diagnosed with APL

A total of 25 patients initially diagnosed with APL, 17 males and 8 females (mean age, 36.4±10.3 years; range, 19–64 years; Table I), were included in the study. Patients were followed up for a median of four years (range, 0.5–13 years) following their initial treatment (data not shown). According to the classification system by Sanz *et al*(4), four patients (16.0%) were at low risk, 12 (48.0%) were at intermediate risk and nine (36.0%) were at high risk of ALP relapse (Table I). All patients were previously administered with ATRA and low-dose cytotoxic agents during initial induction therapy, and 16 patients (64.0%) received adjuvant treatment with ATO or up-titrated ATS. Thirteen patients (52.0%) received intrathecal chemotherapy for the treatment of central nervous system (CNS) involvement at the time of initial diagnosis, and all patients received prophylactic intrathecal chemotherapy following CR (data not shown).

### Clinical characteristics of relapsed patients with APL

The first relapse occurred at a median of 17 months (range, 5–84 months) following initial treatment (Table I). Relapses involved bone marrow in 19 patients (76.0%), the CNS alone in one patient (4.0%), molecular relapse in one patient (4.0%) and bone marrow/CNS (complete relapse) in four patients (16.0%) (Table II). The relapsed patients with APL showed a median WBC count of 4.8×10^9^/l (range, 1.3–144.2×10^9^/l), a median hemoglobin level of 125.9±29.4 g/l (range, 63.9–182 g/l) and a median platelet count of 140.0×10^9^/l (range, 9.0–266.0×10^9^/l) (Table I). First relapse data is presented in comparison with initial clinical values in Table I and in full detail in Table II.

### Re-induction therapy selection and efficacy

**Figure 1:**
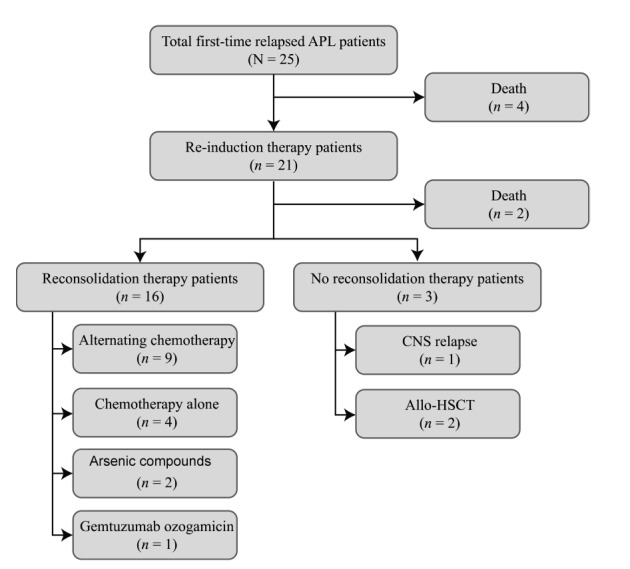
Flowchart of patient treatment and outcome data. APL, acute promyelocytic leukemia; CNS, central nervous system; allo-HSCT, allogeneic hematopoietic stem cell transplantation.

Four relapsed patients with APL succumbed to the disease before re-induction therapy. For re-induction therapy, patients were treated with either ATRA + arsenic compound combination therapy (16/25, 64.0%), salvage chemotherapy with HA (2/25, 8.0%), salvage chemotherapy with HAD (1/25, 4.0%), gemtuzumab ozogamicin (for the single case of molecular relapse; 1/25, 4.0%) or intrathecal chemotherapy (for the single case of isolated CNS relapse; 1/25, 4.0%) (Table II). Two of those who did not survive (8.0%) were treated with ATRA + arsenic compound re-induction therapy. Only one (4.0%) patient recovered completely from isolated CNS relapse following intrathecal chemotherapy, remaining disease-free for 13 years. CR was also observed following ATRA + arsenic compound therapy (10/25, 40.0%), chemotherapy (3/25, 12.0%)and targeted therapy (1/25, 4.0%); and non-remission (NR) following ATRA + arsenic compounds (4/25, 16%) (Table II). Fig. 1 details the treatment and outcomes of the patients.

### Reconsolidation therapy and survival

Among the 19 surviving patients (76.0%), complete recovery from CNS relapse following intrathecal chemotherapy occurred in one patient (1/19, 5.3%); one patient (5.3%) treated with allo-HSCT following secondary CR remained disease-free at the end of the study period; two patients (10.5%) remained in CR following remission re-induction; secondary bone marrow and CNS relapse occurred in 14 patients (73.7%) and one patient (5.3%), respectively. The rate of secondary relapse was 78.9% (15/19) (Table II).

Among the 19 surviving patients (76.0%), complete recovery from CNS relapse following intrathecal chemotherapy occurred in one patient (1/25, 4.0%); one patient (4.0%) treated with allo-HSCT following secondary CR remained disease-free at the end of the study period; two patients (8.0%) remained in CR following remission re-induction; and secondary bone marrow and CNS relapse occurred in 14 patients (56.0%) and one patient (4.0%), respectively. The rate of secondary relapse was 78.9% (15/19) (Table II).

### Toxic effects of ATRA + arsenic compound re-induction therapy

Adverse events were observed in all ATRA + arsenic compound re-induction therapy patients. Of the 16 surviving patients, six (6/16, 37.5%) exhibited no treatment-emergent toxicity, six (6/16, 37.5%) exhibited grade 1–2 hepatotoxicity, two (2/16, 12.5%) exhibited grade 3–4 bone marrow suppression and two (2/16, 12.5%) exhibited treatment-related mortality. Salvage chemotherapy was poorly tolerated in all three patients receiving chemotherapy for re-induction, with grades 3–4 bone marrow suppression. No toxicity was reported in the two patients that received isolated intrathecal chemotherapy or gemtuzumab ozogamicin (Table II).

## Discussion

The present study demonstrated that ATRA + arsenic compound-based combination therapy was effective in re-inducing morphological remission in relapsed patients with APL with previous exposure to ATRA + arsenic compounds; however, these patients remained subject to low molecular remission rates and at high risk of secondary relapse. Notably, allo-HSCT yielded good re-induction results, suggesting that early allo-HSCT should be more carefully explored as a therapeutic option for re-inducing morphological remission in relapsed patients with APL with previous exposure to ATRA + arsenic compounds.

Combinations of ATRA and chemotherapy have been widely accepted as front-line treatments for the majority of relapsed patients with APL (12). In the present study, the overall good results produced by ATRA + arsenic compounds, leading to remission in the majority of patients with APL, are consistent with a previous study that recommends this treatment for salvage patients with APL or those that have previously received ATRA-based combination therapies (16). Furthermore, arsenic compound monotherapy has been reported to effectively re-induce molecular remission in 80–90% of relapsed patients with APL, making it useful as both an initial induction and consolidation treatment in high-risk patients (16). Breccia *et al*(13) reported that prolonged ATO-based salvage therapy achieved a high remission rate in relapsed ATO-naïve patients without requiring HSCT. Cumulatively, the results of the current study and the findings of these previous studies are in agreement and indicate that morphological remission in relapsed patients with APL with previous exposure to ATRA + arsenic compounds may be efficiently obtained using ATRA + arsenic compound-based combination therapy. Notably, these findings are generally consistent with the broader guidelines published by the National Comprehensive Cancer Network in 2011 (20).

In the patient cohort of the present study, APL relapse occurred at a median of 17 months after initial reports of CR, a relapse time significantly earlier than that reported by Thirugnanam *et al* (20.3 months) (14). A high remission re-induction rate was achieved in patients in the current study following ATRA + arsenic compound-based salvage treatment with or without chemotherapy, although these patients had previous exposure to ATRA + arsenic compounds. Furthermore, alternative chemotherapy was generally effective; however, these patients often experienced secondary relapse and the duration of remission was relatively short (median, 15 months). By contrast, Thirugnanam *et al*(14) reported a CR rate of 93% in relapsed patients with APL with previous exposure to ATO monotherapy following ATRA + arsenic compound-based salvage treatment with or without anthracycline-based chemotherapy. Furthermore, these patients with APL only received ATRA as salvage treatment for relapse, rather than induction or maintenance therapy for initial treatment (14); whereas, the majority of patients in the current study received arsenic compounds induction and maintenance therapies as well. This may contribute to discrepancies between the findings of the current study and those of Thirugnanam *et al*(14).

The current results indicated a high frequency of extra-medullary relapse, with the CNS involved in the relapse of approximately one fifth of current patients, and this was inconsistent with a previous study in which only 8% of patients exhibited relapse in the CNS (14). Furthermore, the majority of patients in the present study with CNS relapse also exhibited relapse in the bone marrow. As ATRA upregulates the expression of adhesion molecules, such as CD11b, CD13 and CD56, on ALP cell surfaces (21), and concomitantly stimulates the secretion of interleukin-1 (22), ATRA promotes endothelial expression of vascular cell adhesion molecule 1 and intercellular adhesion molecule 1 (23). As a result, patients with APL exposed to ATRA are more likely to exhibit relapse involving the CNS or even the pseudotumor cerebri (4), which may explain the occurrence of relapse in the CNS in the patients of the present study. Additionally, granulocytosis may independently contribute to CNS involvement during ALP relapse (24), further raising the risk of relapse involving the CNS.

Only a small percentage of the patients in the current study achieved molecular remission, suggesting that relapsed patients with APL previously exposed to ATRA + arsenic compound-based combination treatments were at very high risk of secondary relapse. Notably, all the patients previously treated with ATRA + arsenic compounds combined with chemotherapy exhibited relapse following auto-HSCT. Therefore, auto-HSCT may not be suitable for these patients, despite general recommendations that auto-HSCT rather than allo-HSCT is optimal for use in patients with APL who initially achieve CR following primary relapse (25). By contrast, allo-HSCT should be considered in relapsed patients that exhibit a relatively short duration of remission or do not achieve molecular remission, preferably following the secondary rather than tertiary morphological CR episode (26).

As all the patients included in the current study were exposed to ATRA, further comparative studies are required to identify whether ATRA is involved in the development of CNS relapse and by which mechanisms this action may occur. Additionally, variations in dosage and treatment duration must be considered, which may contribute to relapse occurrence based on yet undetermined risk factors. Furthermore, the sample size of the present study is relatively small and, thus, may not be fully representative of broader APL patient populations.

ATRA + arsenic compound-based salvage treatments with or without chemotherapy are effective agents for re-inducing complete remission in relapsed patients with APL previously exposed to combined ATRA + arsenic compound therapies. Molecular remission, however, is relatively rare following such salvage treatment in relapsed patients with APL, and the vast majority of these patients will exhibit secondary relapse. Furthermore, the findings of the present study suggest that auto-HSCT may be unsuitable for use in relapsed patients with APL who are at high risk of secondary relapse, with early allo-HSCT yielding a more notable beneficial survival benefit. Large-scale comparative studies, however, will be required to fully elucidate this correlation.

## Figures and Tables

**Table I:** Demographic and clinical characteristics of patients with acute promyelocytic leukemia at the time of initial diagnosis and first relapse.

	Value	
	
Characteristics	Initial diagnosis	Relapse
Gender, n (%)		
Male	17 (68.0)	
Female	8 (32.0)	
Age, years	36.4±10.3	
WBC count, 10^9^/l^a^	7.8±16.6	4.8±2.7
Hemoglobin, g/dl^a^	99.0±52.0	125.9±29.4
DIC, n (%)	12 (48.0)	9 (36.0)
CNS leukemia, n (%)	13 (52.0)	11 (44.0)
Sanz risk category^b^, n (%)		
Low risk	4 (16.0)	
Intermediate risk	12 (48.0)	
High risk	9 (36.0)	
Platelet count, 10^9^/l^a^	28.2±33.0	140.0±139.0
Chromosome aberration, n (%)	7 (28.0)	
Equiarm 17q	1 (4.0)	
+2p−, −4, inv(14), 22p+, +mar	1 (4.0)	
+8	1 (4.0)	
1p+, 16q+	1 (4.0)	
7q−	1 (4.0)	
16p+, 14q−	1 (4.0)	
11p+, 17p−, −14, −20, ace^a^2	1 (4.0)	
CD56 expression, n (%)		
Negative	6 (24.0)	
Positive	3 (12.0)	
Unknown	16 (64.0)	
CD117 expression, n (%)		
Negative	1 (4.0)	
Positive	11 (44.0)	
Unknown	13 (52.0)	
Time to relapse, months^a^	17.0±17.0	15.0±17.0

Patients were initially treated with all-*trans* retinoic acid and arsenic compound-based combined therapies. Data are presented as the mean ± SD unless otherwise specified.

a The median ± interquartile range.

b Category taken from (4).

WBC, white blood cell; DIC, disseminated intravascular coagulation; CNS, central nervous system.

**Table II:** Clinical characteristics of patients with acute promyelocytic leukemia at the time of first relapse.

Characteristics	n (%)
Relapse type	
Bone marrow	19 (76.0)
CNS alone	1 (4.0)
Molecular	1 (4.0)
Bone marrow/CNS	4 (16.0)
First relapse induced	
ATRA + arsenic compounds	16 (64.0)
ED	4 (16.0)
GO	1 (4.0)
Salvage chemotherapy	3 (12.0)
Intrathecal chemotherapy	1 (4.0)
Induced side effects	
ED	6 (24.0)
Grade 1 hepatotoxicity	2 (8.0)
Grade 2 hepatotoxicity	4 (16.0)
Grade 3 bone marrow suppression	4 (16.0)
Grade 4 bone marrow suppression	1 (4.0)
None	8 (32.0)
First recurrence consolidate	
ATRA + arsenic compounds	
with alternating chemotherapy	9 (36.0)
ED	6 (24.0)
GO	1 (4.0)
Chemotherapy alone	4 (16.0)
Arsenic compounds	2 (8.0)
None	3 (12.0)
First relapse treatment efficacy	
CR	15 (60.0)
ED	6 (24.0)
NR	4 (16.0)
Second relapse	
ED	6 (24.0)
Non-relapse	4 (16.0)
Relapse	15 (60.0)
Third relapse	
ED	11 (44.0)
Non-relapse	6 (24.0)
Died of allo-HSCT	2 (8.0)
Relapse	6 (24.0)
Current Status	
CR	8 (32.0)
Death	17 (68.0)

Patients were initially treated with all-*trans* retinoic acid and arsenic compound-based combined therapies. CNS, central nervous system; ATRA, all-*trans* retinoic acid; ED, early death; GO, gemtuzumab ozogamicin; CR, complete remission; NR, non-remission; allo-HSCT: allogeneic hematopoietic stem cell transplantation.

## References

[1] Douer D, Preston-Martin S, Chang E, Nichols PW, Watkins KJ, Levine AM (1996). High frequency of acute promyelocytic leukemia among Latinos with acute myeloid leukemia. Blood.

[2] Vickers M, Jackson G, Taylor P (2000). The incidence of acute promyelocytic leukemia appears constant over most of a human lifespan, implying only one rate limiting mutation. Leukemia.

[3] Kelaidi C, Chevret S, De Botton S (2009). Improved outcome of acute promyelocytic leukemia with high WBC counts over the last 15 years: the European APL Group experience. J Clin Oncol.

[4] Sanz MA, Grimwade D, Tallman MS (2009). Management of acute promyelocytic leukemia: recommendations from an expert panel on behalf of the European LeukemiaNet. Blood.

[5] Degos L, Zhen YW (2001). All trans retinoic acid in acute promyelocytic leukemia. Oncogene.

[6] Lo-Coco F, Avvisati G, Vignetti M (2010). Front-line treatment of acute promyelocytic leukemia with AIDA induction followed by risk-adapted consolidation for adults younger than 61 years: results of the AIDA-2000 trial of the GIMEMA Group. Blood.

[7] Fu S, Consoli U, Hanania EG (1995). PML/RARalpha, a fusion protein in acute promyelocytic leukemia, prevents growth factor withdrawal-induced apoptosis in TF-1 cells. Clin Cancer Res.

[8] de The H, Chomienne C, Lanotte M, Degos L, Dejean A (1990). The t(15;17) translocation of acute promyelocytic leukaemia fuses the retinoic acid receptor alpha gene to a novel transcribed locus. Nature.

[9] Fenaux P, Chastang C, Chevret S, The European APL Group (1999). A randomized comparison of all transretinoic acid (ATRA) followed by chemotherapy and ATRA plus chemotherapy and the role of maintenance therapy in newly diagnosed acute promyelocytic leukemia. Blood.

[10] Callens C, Chevret S, Cayuela JM (2005). Prognostic implication of FLT3 and Ras gene mutations in patients with acute promyelocytic leukemia (APL): a retrospective study from the European APL Group. Leukemia.

[11] Tallman MS, Andersen JW, Schiffer CA (2002). All-trans retinoic acid in acute promyelocytic leukemia: long-term outcome and prognostic factor analysis from the North American Intergroup protocol. Blood.

[12] Asou N, Kishimoto Y, Kiyoi H (2007). A randomized study with or without intensified maintenance chemotherapy in patients with acute promyelocytic leukemia who have become negative for PML-RARalpha transcript after consolidation therapy: the Japan Adult Leukemia Study Group (JALSG) APL97 study. Blood.

[13] Breccia M, Cicconi L, Minotti C (2011). Efficacy of prolonged therapy with combined arsenic trioxide and ATRA for relapse of acute promyelocytic leukemia. Haematologica.

[14] Thirugnanam R, George B, Chendamarai E (2009). Comparison of clinical outcomes of patients with relapsed acute promyelocytic leukemia induced with arsenic trioxide and consolidated with either an autologous stem cell transplant or an arsenic trioxide-based regimen. Biol Blood Marrow Transplant.

[15] Ghavamzadeh A, Alimoghaddam K, Ghaffari SH (2006). Treatment of acute promyelocytic leukemia with arsenic trioxide without ATRA and/or chemotherapy. Ann Oncol.

[16] Ravandi F, Estey E, Jones D (2009). Effective treatment of acute promyelocytic leukemia with all-trans-retinoic acid, arsenic trioxide, and gemtuzumab ozogamicin. J Clin Oncol.

[17] American Cancer Society Publications Leukemia-Acute Myeloid (Myelogenous): How is it classified?.

[18] de Botton S, Sanz MA, Chevret S (2006). Extramedullary relapse in acute promyelocytic leukemia treated with all-trans retinoic acid and chemotherapy. Leukemia.

[19] National Cancer Institute (1999). Cancer therapy evaluation program: Common toxicity criteria manual.

[20] National Comprehensive Cancer Network (2011). Clinical practice guidelines in oncology: Acute myeloid leukemia.

[21] Nagai S, Takahashi T, Kurokawa M (2009). Beneficial and adverse effects of molecularly targeted therapies for acute promyelocytic leukemia in central nervous system. CNS Neurol Disord Drug Targets.

[22] Machner B, Neppert B, Paulsen M, Hofmann C, Sander T, Helmchen C (2008). Pseudotumor cerebri as a reversible side effect of all-trans retinoic acid treatment in acute promyelocytic leukaemia. Eur J Neurol.

[23] de Botton S, Fawaz A, Chevret S (2005). Autologous and allogeneic stem-cell transplantation as salvage treatment of acute promyelocytic leukemia initially treated with all-trans-retinoic acid: a retrospective analysis of the European acute promyelocytic leukemia group. J Clin Oncol.

[24] O’Brien S, Kantarjian HM, Keating M (1989). Association of granulocytosis with poor prognosis in patients with acute myelogenous leukemia and translocation of chromosomes 8 and 21. J Clin Oncol.

[25] Kohno A, Morishita Y, Iida H (2008). Hematopoietic stem cell transplantation for acute promyelocytic leukemia in second or third complete remission: a retrospective analysis in the Nagoya Blood and Marrow Transplantation Group. Int J Hematol.

[26] Bourquin JP, Thornley I, Neuberg D (2004). Favorable outcome of allogeneic hematopoietic stem cell transplantation for relapsed or refractory acute promyelocytic leukemia in childhood. Bone Marrow Transplant.

